# Fifteen years of hoarseness - case report of a rare laryngeal schwannoma

**DOI:** 10.5935/1808-8694.20130068

**Published:** 2015-10-04

**Authors:** Paulo Saraceni Neto, Anelise Abrahão Salge Prata, José Eduardo de Sá Pedroso, Luciano Rodrigues Neves, Osíris do Brasil

**Affiliations:** a MD. Otorhinolaryngologist. Medical Residency and Fellowship by the Department of Otorhinolaryngology and Head and Neck Surgery - Paulista School of Medicine - Federal University of São Paulo (UNIFESP).; b MD, ENT, PhD in ENT by the Department of ENT-HNS at UNIFESP/Paulista Medical School (MD, ENT, PhD in ENT by the Department of ENT-HNS at UNIFESP/Paulista Medical School).

**Keywords:** cranial nerve neoplasms, dysphonia, laryngeal neoplasms, neuroma, Schwann cells

## INTRODUCTION

Schwannomas are benign neurogenic tumors that originate in the Schwann sheath of any cranial or spinal nerve, minus those belonging to the optic and olfactory tracts^1^, ^2^.

Laryngeal schwannomas are rarely found, and account for 0.1% to 1.5% of all benign tumors of the larynx^1^.

Laryngeal schwannomas may cause dysphonia, vocal fold fixation, and even airway obstruction, depending on their size and location^1^, ^2^, ^3^, ^4^, ^5^, ^6^.

## CASE REPORT

J.E.S., 25, male, complained of hoarseness persisting for 15 years and growing more intense in the last two years. He also alluded to pharyngeal globus sensation, breathlessness when in a supine position, and dyspnea when under moderate physical strain.

The patient reported that he did not drink or smoke, and that he had not been hospitalized or undergone surgery previously.

Physical examination revealed the patient had stridor when inhaling and used accessory muscles during ventilation.

**Figure 1:**
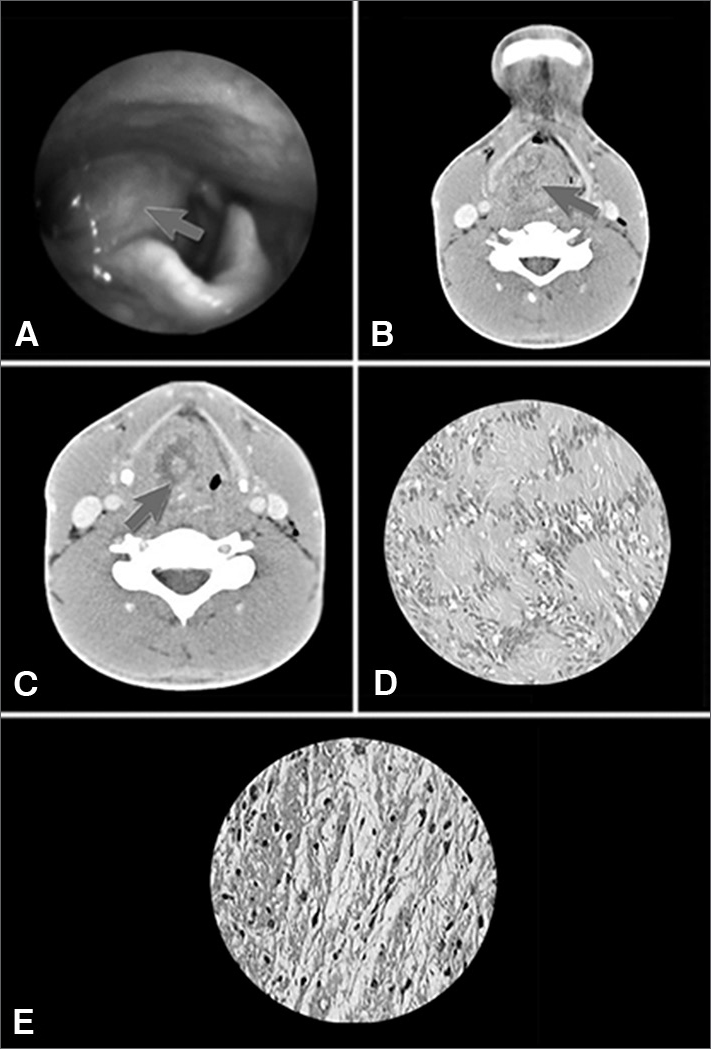
A: Tumor in the right aryepiglottic fold obliterating the ipsilateral piriformis sinus (arrow); B: Heterogeneous image obliterating the pharyngeal lumen (CT scan of the supraglottis - axial view - arrow); C: Tumor obstructing the glottis and preventing the proper identification of the vocal folds (CT scan of the glottis - axial view - arrow); D: Presence of Antoni A areas in histology examination (400x magnification); E: Presence of Antoni B areas in histology examination (400x magnification).

Indirect laryngoscopy showed he had a smooth submucosal tumor located in his right aryepiglottic fold, obstructing the right piriformis recess and preventing proper visualization of the ipsilateral vocal fold and the glottic hiatus (Figure 1A).

Neck CT scans unveiled a round heterogeneous tumor (five centimeters in its larger diameter) partially obliterating the larynx and extending from the right aryepiglottic fold to the ipsilateral vocal fold (Figures 1B & 1C).

The patient underwent emergency tracheostomy and suspension microlaryngoscopy for an incisional biopsy.

The pathologist's report indicated that the patient had a schwannoma, and it was decided that the tumor would be removed through a laryngofissure procedure. The procedure was carried out successfully, the entire tumor was removed and the mucosa and adjacent cartilages were were preserved.

Four months after surgery the patient had no respiratory or swallowing complaints, but was still dysphonic due to a right vocal fold paresis in the paramedian position.

## DISCUSSION

Laryngeal schwannomas are rare benign tumors described for the first time by Schwanck in 1925^3^.

The characteristic finding in laryngoscopy is the presence of a round smooth pinkish submucosal tumor emerging from the vestibular fold or the aryepiglottic fold, possibly obstructing the larynx depending on how much it has grown^3^, ^4^.

Growth is usually slow, and symptoms (roughness, pharyngeal globus, dysphagia, dyspnea, and stridor) tend to be related to the size and site of the tumor^4^.

The dyspnea while in a supine position reported by the patient had been previously described and associated with supraglottic obstruction^4^. Patients may be eligible for emergency tracheostomy depending on the degree of obstruction, as complications such as asphyxia and death may occur^4^, ^5^.

Large laryngeal schwannomas as the one presented in this paper are rare. Only one case of a schwannoma of a larger size has been reported^6^.

The differential diagnosis for neurinoma includes neurofibroma, laryngeal cyst, laryngocele, laryngopyocele, and benign laryngeal tumors.

Imaging is required to determine the characteristics of the tumor and its extension. Absence of infiltration, round tumors, location medially to the thyroid cartilage, and preserved thyroid cartilage are traits seen in laryngeal schwannomas^3^.

Definitive diagnosis is done through histology testing. Schwann cells cluster together and form a block of cells with their nuclei aligned in palisades (Antoni A pattern) or arrange themselves in sparsely cellular areas amidst myxoid matrix and edema (Antoni B pattern)^1^ (Figures 1D & 1E).

The treatment of choice, as this is a benign tumor, is surgical removal with preservation of laryngeal function. Endoscopic excision is possible for small tumors. Open surgery is preferred when tumor involvement is more extensive.

## CLOSING REMARKS

This is a case report of a laryngeal tumor that had been evolving for 15 years in a patient with complaints of airway obstruction, which culminated with the discovery of a large tumor in the subjects larynx.
